# Atmospheric Turbulence Degraded Video Restoration with Recurrent GAN (ATVR-GAN)

**DOI:** 10.3390/s23218815

**Published:** 2023-10-30

**Authors:** Bar Ettedgui, Yitzhak Yitzhaky

**Affiliations:** 1 Department of Electrical Engineering, Tel Aviv University, Tel Aviv 69978, Israel; barettedgui@mail.tau.ac.il; 2 Department of Electro Optics Engineering, School of Electrical and Computer Engineering, Ben Gurion University of the Negev, Be’er Sheva 8410501, Israel

**Keywords:** atmospheric turbulence, video restoration, GAN, RNN, CNN, optical flow

## Abstract

Atmospheric turbulence (AT) can change the path and direction of light during video capturing of a target in space due to the random motion of the turbulent medium, a phenomenon that is most noticeable when shooting videos at long ranges, resulting in severe video dynamic distortion and blur. To mitigate geometric distortion and reduce spatially and temporally varying blur, we propose a novel Atmospheric Turbulence Video Restoration Generative Adversarial Network (ATVR-GAN) with a specialized Recurrent Neural Network (RNN) generator, which is trained to predict the scene’s turbulent optical flow (OF) field and utilizes a recurrent structure to catch both spatial and temporal dependencies. The new architecture is trained using a newly combined loss function that counts for the spatiotemporal distortions, specifically tailored to the AT problem. Our network was tested on synthetic and real imaging data and compared against leading algorithms in the field of AT mitigation and image restoration. The proposed method outperformed these methods for both synthetic and real data examined.

## 1. Introduction

Long-range imaging is deeply affected by the atmospheric medium, which causes dynamic deformations and blur in the resulting video. The effects of atmospheric turbulence are caused due to shifts and changes in density, temperature, and humidity, which directly affect the reflective index of the optical medium and cause said degradations. Hence, the need for the reconstruction of degraded videos that have suffered atmospheric turbulence is beneficial if one wishes to engage in higher tasks such as classification [1], object detection, tracking [2,3], etc.

To mitigate the effect of atmospheric turbulence, many image-processing-based methods have been proposed over the years. These methods can be divided into three main approaches: image-to-image methods [4,5,6,7,8,9], which were the main study subject for recent AT reconstruction research using both classical and deep learning-based algorithms; sequence-to-single image methods [10,11,12,13,14,15], which assume that the scene and position of the camera are fixed while using multi-frame inputs in order to produce a single good image and finally the least studied subject over the previous decade; and video-to-video methods [16,17,18,19] that focus on video AT mitigation, where the input to the model is a frame sequence and the output is the restored frame sequence with mitigated AT deformation and blur.

The reconstruction of a video degraded due to atmospheric turbulence is of an ill-posed nature and can be mathematically modeled in the following way, as used by [10]. We define {A} as the set of all the observed frames, $f_{t\in \left[0,T\right]}^{A}$, and {B} as the set of all the real undistorted AT frames $f_{t\in \left[0,T\right]}^{B}$, which we ideally want to recover from the observed frames. Next, we define ${Dist}_{t}$ as the geometric distortion caused by angle-of-arrival fluctuations caused by a turbulent atmosphere at time t, a blurring kernel ${Blur}_{t}$ at time t, which is commonly assumed to be stationary for short periods with respect to the geometric distortion [2], and $n_{t}$, which represents some additive noise at time t.

(1) $$f_{t}^{A}={Blur}_{t}\left({Dist}_{t}\left(f_{t}^{B}\right)\right)+n_{t}$$

Recently, many learning-based algorithms have been proposed to tackle problems of a similar nature, like super-resolution and unpaired video-to-video translation, yielding state-of-the-art results, such as Recycle-GAN [20] and iSeeBetter [21]. These great breakthroughs rely on cutting-edge deep learning algorithms, including CycleGAN [22], optical flow estimation algorithms, such as FlowNet [23], and RNN algorithms, such as ConvLSTM [24], which have made it possible for the development of these new methods. 

Methods for AT image restoration can be divided into two main approaches: image-to-image and sequence-to-image. The former is currently the main active research approach, combining innovative deep learning techniques and blind deconvolution methods, which rely on mathematical and physical modeling of the turbulence degradation effect. Recently, the authors of [5] proposed an iterative algorithm called BATUD, which is based on a physical model for the modulation transfer function of the imaging system and the impact of the turbulence using the Fried kernel. The proposed method is used to perform deconvolution and then estimate the Fried kernel [25] by jointly relying on a Gaussian Mixture Model (GMM) prior to natural image patches and regularizing with the square Euclidean norm of the Fried kernel. X. Bai et al. [6] conducted a comparative research between Fully Convolutional Networks (FCNs) and conditional GAN (CGAN) with perceptual loss [26] and adversarial loss [27], revealing that these networks outperform classical methods while restoring high-frequency details and textures and suppressing noise effectively. O. Chen et al. [7] focused their research on the imaging of outer space targets, combining FCN with dilated convolutions for denoising before propagating through an asymmetric U-net [28] with transposed convolution. C. P. Lau et al. [8] tackled the task of face image restoration under AT, with a three Wasserstein-GAN (WGAN) [29] with a gradient penalty two pathway architecture for deblurring and deconstruction, respectively, along with a fusion network, utilizing both perceptual [26] and adversarial loss [26] functions while using a PatchGAN [22] architecture for the discriminators and a DeblurGAN-based [30] generator architecture. R. Yasarla and V. M. Patel [8] proposed AT-Net, a deep CNN that combines two networks. One assesses the degradation of the AT on the given image by using Monte Carlo dropouts to estimate the epistemic uncertainty and use it as a prior measure of the AT degradation at each pixel, and then a second network is used to estimate the clean image.

Sequence-to-image methods contain more information about the given scene but have to overcome temporal problems like dynamic scenes or moving objects. Nonetheless, in recent years, several studies have been performed using this approach. Usually, the process of sequence-to-image transformation involves a reference frame, which is of sharp and undistorted quality that can be referred to as a “lucky image”, followed by a registration step, where all other frames are registered to the “lucky image” under some criteria to produce a single good image. However, statistically, there is no guarantee for such a “lucky image” to even exist, particularly in regular horizontal imaging through the atmosphere. X. Zhu and P. Milanfar [10] suggested using a B-spline-based non-rigid image registration algorithm to register each observed frame with respect to a reference frame while introducing a symmetry constraint for accuracy enhancement. In the reconstruction part, they used an L1 norm and bilateral total variation (BTV) regularization term to enhance image quality. In a sequel work [11] a few years later, the authors used a B-spline-based non-rigid image registration and, for the second stage, they proposed the use of a blind deconvolution algorithm to deblur the fused image. N. Anantrasirichai et al. [12] proposed a method termed CLEAR, which introduced a new reference frame creation technique through the selection of regions from different frames based on a quality metric followed by fusion at the feature level by using Dual-Tree Complex Wavelet Transform. C. P. Lau et al. [13] proposed optimizing a cost function, including criteria for sharpness, distortion and number of sampled frames, for sampling “good” frames from which a sharp image is created via the temporal mean of the sampled “good” frames. Afterward, a stabilization stage was proposed in order to remove geometric deformations by wrapping each frame using a suitable deformation field calculated with large displacement optical flow while using Robust Principal Component Analysis (RPCA) for outlier suppression. Finally, registration and image fusion steps were carried using an image fusion scheme followed by deconvolution to deblur the finite image. Later that year, the same authors published [14], where several variational models were studied to simultaneously determine the optimal subsampling of frames and the extraction of a clear image, afterwards a registration step is carried out to register each frame to a reference image, and then the turbulent deformation matrix can be estimated and a sharp image can be reconstructed. Recently, Z. Mao et al. [15] proposed an averaging method to construct a reference frame and a lucky region fusion method followed by a blind deconvolution step that showed superior but close results to CLEAR [12].

The video restoration of AT-degraded videos has been the least studied subject in recent years. It is considered to be more complicated than single-image restoration tasks, for one has to consider not just blur and geometric distortion in one frame but in the whole sequence of frames while taking into account object movement and temporal and spatial movements in both the scene and even the camera. When concerning real-world applications, like long-range video object tracking and super-resolution video, one must first tackle the task at hand in order to achieve applicable results. The authors of [16] proposed an adaptive control grid interpolation method for the case of a static camera with dynamic scenes by first performing a bilinear interpolation to increase the spatial resolution. Next, a calculation of a high-resolution dynamic motion vector field is derived from the video data using a minimization process, assuming that the AT disturbance is quasi-periodic, a base frame is achieved, and the motion field is used to correct AT distortion. S. Gepshtein et al. [17] used a Differential Elastic Image registration method by generating a good reference image using a rank smoothing filter to create a static image of the scene, eliminating any moving parts. Next, a motion field is achieved via the registration of the spatial neighborhood of each pixel to the reference image, which is then used to eliminate AT geometric distortions from static parts of the scene. To deal with moving objects, an error function was computed, providing a large score for moving objects with respect to AT distortion, which is used to truncate the motion vectors of those objects. Y. Lou et al. [18] proposed applying a Sobolev gradient method to sharpen individual frames and mitigate the temporal distortions via the Laplace operator. Recently N. Anantrasirichai [19] suggested the use of complex-valued convolutions on the basis that it captures phase information from the atmospheric turbulence better than real-valued CNNs. The results shown in the paper outperformed a regular U-Net [28] by a small difference, where no special attention was given to the AT problem.

Motivated by the recent success in RNNs and GANs, we propose a novel Atmospheric Turbulence Video Restoration Generative Adversarial Network (ATVR-GAN) with the following innovations intended for AT video degradation recovery:

- A novel RNN generator architecture which includes:
  o A preprocessing stage dedicated to acquiring an initial estimation of the turbulence flow.
  o Customized memory cells specifically aimed for the propagation of AT knowledge across timestamps.
  o A post-processing stage aimed at producing both temporal and spatial updates for the network’s knowledge given the scene and turbulence predictions.
  o An AT prediction sub-network, trained to predict the current AT optical flow map by learning from the posterior knowledge of the scene.
- A novel use of the following combined loss function integrating perceptual loss [26], adversarial loss [27], total variation (TV) loss [31], optical flow loss and AT loss.

## 2. Method

### 2.1. Problem Definition

We argue that our task can be modeled as a domain transfer from an AT domain {A} to an AT undistorted domain {B}. As such, we propose a novel architecture for AT video restoration combining deep learning building blocks from GANs and RNNs and a new loss function for our model to optimize by considering spatial and temporal constraints, as well as the nature of AT disturbances, which can manifest as blur and spatial dispositions. The goal is to mitigate AT effects in video frames and, by that, transfer them into a domain where they appear to be sharper and temporally more coherent. To achieve this goal, we set some assumptions to help define our problem:

- We focus our research on ground-level imaging under anisoplanatic atmospheric turbulence, where the medium is assumed to be of the same level along the path of propagation [32] and where the size of the objects is relatively small with respect to propagation length.
- The video is taken from a constant position, which may move radially in yaw and pitch angles but not axially. The justification for such a constraint is due to the prime intended use of our algorithm, which is intended for surveillance missions or long-distance capturing under relatively high zoom ratios for several to tens of kilometers where movements in yaw, pitch and zoom are most relevant but axial movements are not.
- The scene may alter and contain dynamic objects and zoom in/out scenarios.

### 2.2. Algorithm and Arcitecture

**Figure 1:**
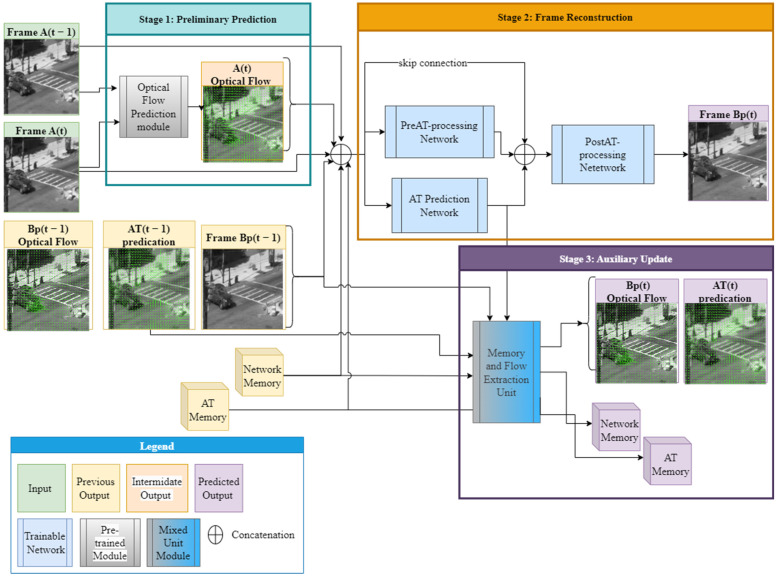
ATVR generator arcithecture.

The ATVR-GAN model was designed to capture both spatial and temporal features in the received turbulent scene while resolving atmospheric turbulence disturbance, which, as explained before, manifests mainly as blur and dispositions. Our model is a GAN based on a novel RNN generator architecture, as shown in Figure 1, while harnessing a proven discriminator architecture from PatchGAN [22], as used in [20,33].

After observing the remarkable achievements in video-to-video translation, particularly recent breakthroughs like Recycle-GAN [20] and iSeeBetter [21], which harness the potential of adversarial loss [26], we were inspired to adopt a GAN-based architecture for our own model. These cutting-edge approaches have demonstrated the ability to generate remarkably realistic results, especially in scenarios where the input data are limited while the output demands intricate details.

Our generator architecture can be seen as being comprised of 3 stages: preliminary flow prediction, frame reconstruction and auxiliary update. The only external input to the network includes the current frame and previous frame, and the external output is the current predicted frame. In the first stage, a prior for the input frames’ AT flow is predicted, which is then concatenated with previous internal and external outputs of the network and inserted into the second stage, which yields the current predicted frame. The last step updates the memory cells and computes other internal outputs to be used in the following time stamp as inputs to the second stage. The three stages are further elaborated in the following paragraphs.

#### 2.2.1. Stage 1: Preliminary Flow Prediction

In the first stage, two distorted frames $f_{t}^{A}$ and $f_{t-1}^{A}$ are used for the initial prediction of a dense optical flow map ${OF}_{t}^{A}$ between the turbulent frames using the GMA [34] method. The resultant flow map is used as the preliminary knowledge of the overall scene’s flow that may include non-turbulence-induced motions (depending on the scene’s dynamics, e.g., moving cars or a static scene where only turbulence-induced movement is present) for the next stage, supplying the model with initial information of the combined scene and AT optical flow.

#### 2.2.2. Stage 2: Frame Reconstruction

**Figure 2:**
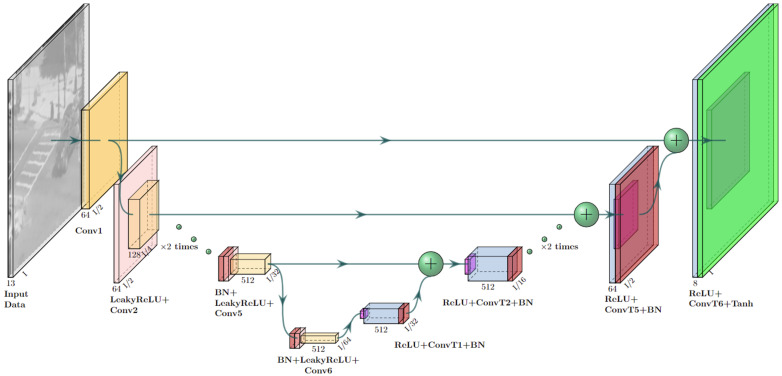
Pre AT-processing network architecture.

**Figure 3:**
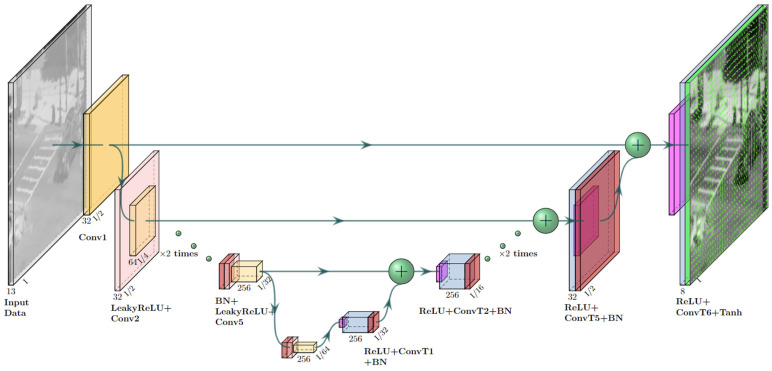
AT OF prediction network architecture.

**Figure 4:**
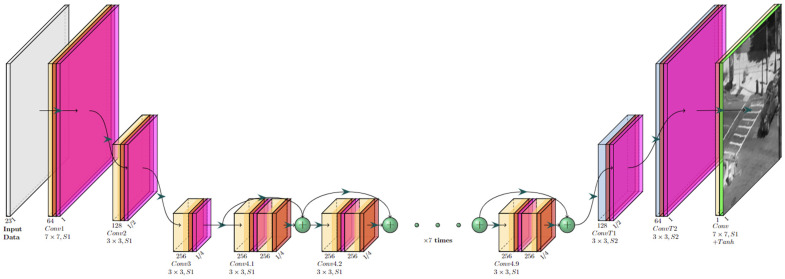
Post AT processing network architecture.

The second stage makes use of the current and previous inputs and outputs from the model and injects them into two networks: the Pre AT-processing Network (Figure 2), which acts as a feature extraction network, and the AT prediction Network (Figure 3), which is trained to predict the current AT optical flow ${\hat{OF}}_{t}^{AT_{expected}}$ induced only by the turbulence effect without non-turbulence motions (such as that of moving objects). From there, the concatenated outputs are inserted into a third Post AT-processing network (Figure 4), which combines all the knowledge from the feature extractor and the predicted AT optical flow and yields the restored frame ${\hat{f}}_{t}^{B}$. 

To that end, two frames, $f_{t}^{A}$ and $f_{t-1}^{A}$, along with the predicted optical flow map ${OF}_{t}^{A}$ from the first stage, are concatenated with previous outputs from the generator at time t−1 and inserted parallel to the Pre AT-processing Network and AT prediction Network.

The previous outputs include: the previously restored frame ${\hat{f}}_{t-1}^{B}$, previously calculated optical flow map between ${\hat{f}}_{t-1}^{B}$ and ${\hat{f}}_{t-2}^{B}$: ${\hat{OF}}_{t-1}^{B}$, previous AT predicted flow map ${\hat{OF}}_{t-1}^{AT}$ and the two auxiliary memory cells. The outputs from said networks are concatenated along with the inputs to the two networks and inserted to the third Post AT-processing network, which produces the reconstructed frame ${\hat{f}}_{t}^{B}$.

The architectures for the Pre AT-processing network, AT Prediction network and Post AT processing network are shown in Figure 2, Figure 3 and Figure 4, respectively. As can be seen from these figures, all our networks are built in an encoder–decoder structure, where the first two (Figure 2 and Figure 3) are based on Unet [18] with a convolution kernel size of 4 × 4 and a combination of Leaky ReLU (pink) with a slope of 0.2 for the encoder and ReLU (magenta) for the decoder. The third network was designed to combine features from previous networks, and to that end, it was constructed to work in a higher spatial resolution than the ones used in the previously mentioned networks and, therefore, equipped with a straightforward convolution stack with batch normalization (red) and ReLU activation applied between two convolutions (the convolution kernels and strides are shown for each layer in Figure 4) and skip connections between blocks for gradient flow.

#### 2.2.3. Stage 3: Auxiliary Update

**Figure 5:**
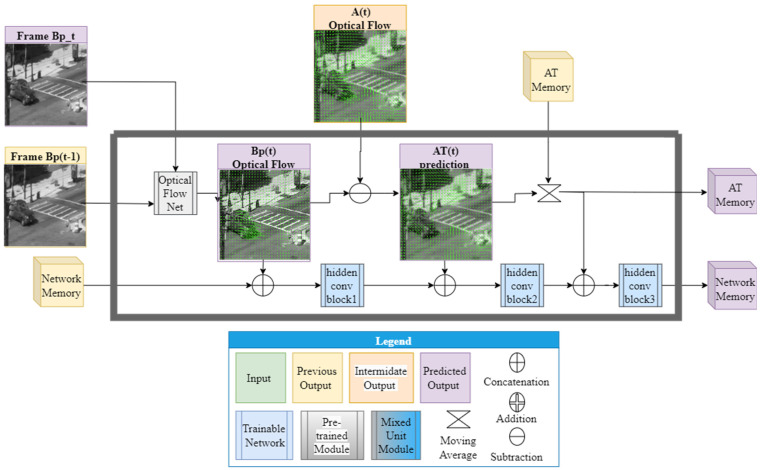
Memory and Flow Extraction (M&F) Unit. The hidden blocks’ architecture is detailed in Table 1.

As shown in Figure 1 and further detailed in Figure 5, the third stage is solely comprised of the Memory and Flow Extraction Unit, which was designed for two main tasks: updating the auxiliary memory cells and calculating the resulting optical flow maps.

The auxiliary memory cells include two dedicated memory cells: the Network Memory cell and the AT Memory cell. The former was designed to provide the network with general recurrent properties by propagating information from different outputs of the network. The latter allows for the utilization of the quasi-periodic nature of the turbulence by aggregating the latest AT flow maps ${\hat{OF}}_{t\in \left[0,t\right]}^{AT}$ using a moving average where α is a hyperparameter (set to 0.7 in our model) that leverages past knowledge versus incorporating new knowledge.

(2) $${\hat{OF}}_{t\in \left[0,t\right]}^{AT}=\alpha \times {\hat{OF}}_{t}^{AT}+(1-\alpha )\times {\hat{OF}}_{t\in \left[0,t-1\right]}^{AT}$$

The auxiliary cells integrate key features across time stamps by extracting knowledge from the newly predicated ${\hat{f}}_{t}^{B},\;{\hat{OF}}_{t}^{B}$ and ${\hat{OF}}_{t}^{AT}$ to update the state of the auxiliary cells and, in doing so, result in recurrent properties in terms of the generator.

**Figure 6:**
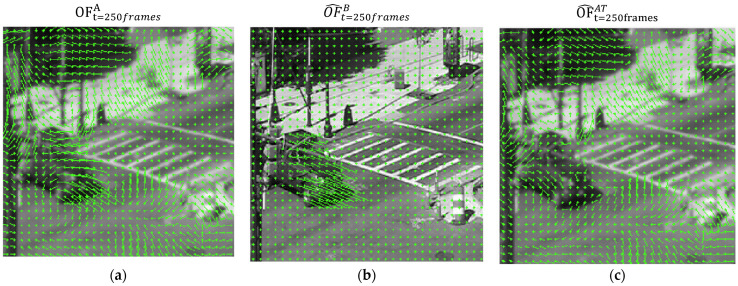
AT memory auxiliary cell integration at t = 250 frames, obtained by adding the subtraction of (**a**) the predicted input OF map $OF_{t}^{A}$ from (**b**) the predicted OF map between the reconstructed frames ${\hat{OF}}_{t}^{B}$, resulting in (**c**), the accumolated OF map ${\hat{OF}}_{t=250frames}^{AT}$. (Vector field is increased by a factor of 8 for visual purposes).

The second task of the M&F unit is to calculate two optical flow maps: one is used for the current optical flow between the predicted restored frames ${\hat{f}}_{t}^{B}$ and ${\hat{f}}_{t-1}^{B}$: ${\hat{OF}}_{t}^{B}$ is used for the calculation of the optical flow maps, as further explained in Section 2.3.5. The second optical flow map is used for a pseudo prediction of the current AT flow map ${\hat{OF}}_{t}^{AT}$, which is calculated by subtracting the ${\hat{OF}}_{t}^{B}$ from ${OF}_{t}^{A}$ (calculated in stage 1), as can be seen in Figure 6. This map in theory counts only for the OF movement caused by the turbulence, excluding movement caused by dynamic objects or camera motion, as demonstrated in Figure 6, where the cars motion is absent from the predicted AT flow map ${\hat{OF}}_{t=250frames}^{AT}$ but is most noticeable in the outputted OF frame ${\hat{OF}}_{t=250frames}^{B}.$

The M&F Unit uses the pretrained OF network GMA [34] that takes the previous predicted frame ${\hat{f}}_{t-1}^{B}$ and current predicted frame ${\hat{f}}_{t-1}^{B}$ and yields the current predicted optical flow map ${\hat{OF}}_{t}^{B}$, which is then used for the calculation of the pseudo AT estimation ${\hat{OF}}_{t}^{AT}$. Finally, the AT auxiliary memory cell is computed by inserting the previous AT memory cell with the current pseudo AT estimation ${\hat{OF}}_{t}^{AT}$ estimation into Equation (2).

To update of the Network Memory auxiliary cell, three convolution blocks are used. The architecture for the Hidden blocks is described in Table 1. Each block is built by integrating the new outputs from the network. First, the previous Network Memory cell, which we empirically set to {256, 256, 1, 2}, is concatenated with the current predicted frame ${\hat{f}}_{t}^{B}$ and inserted to the first convolution block (ConvBlock1). Then, the output from ConvBlock1 is concatenated with ${\hat{OF}}_{t}^{B}$ and inserted into ConvBlock2. Finally, the new Network Memory cell is computed by concatenating the output from ConvBlock2 with the pseudo AT predication ${\hat{OF}}_{t}^{AT}$ and inserting them into the third and final ConvBlock3.

### 2.3. Loss Function

To better address the problem of AT, we needed to compose a loss function that will teach our model how to improve upon both visual and temporal disturbances caused by AT. To do so, we combined five different losses, each designed to tackle different aspects of the problem at hand.

To deal with missing information caused by capturing images under disturbed conditions, we chose Adversarial loss [27] to encourage the network to invent and fill new information where it is scarce or unknown. As our leading engine, we used perceptual loss [26], which uses its learned knowledge from the high-dimensional features of real images to teach the network about the divergence caused by AT-affected features. As a regularization factor for preventing noise output, we used TV loss [31], which encourages the network to produce clean edges and decrease the general noisiness in the image.

Since our focus is on video damaged by AT, we introduced two temporal-based loss functions to ensure coherency between output frames by using OF loss and improve the network’s knowledge of the turbulence flow by introducing AT loss, which teaches the AT Prediction network to predict the current AT flow.

#### 2.3.1. Adversarial Loss

Our network is GAN-based and is comprised of an ATVR generator and a PatchGAN [22] discriminator. The adversarial loss trains both the generator and the discriminator, where the generator learns to produce images with high resemblance to the learned output distribution $\left\{\tilde{B}\right\}$ over the training set, and the discriminator learns the high dimensional features that separate the real images $f_{t}^{B}\in \{B\}$ from the synthetic ones ${\hat{f}}_{t}^{B}\sim G^{\tilde{B}}$, thereby punishing the generator for deviating from the learned output distribution $\left\{\tilde{B}\right\}$ and encouraging it to innovate new information to “trick” the discriminator.

To train the generator and the discriminator, we used the Mean Squared Error (MSE) version [33] of the adversarial loss function, which is more moderate compared to the vanilla log-based [27] version with regards to error magnitude and resulted in more stable training for our GAN model, resulting in fewer mode collapses while training. 

Generator loss $L_{Adversarial}^{G}$ is the result of the MSE between the prediction of the discriminator on a generated frame $G\left(f_{t}^{A}\right)={\hat{f}}_{t}^{B}$ where it is regarded as real (where 1 is real and 0 is fake). The discriminator loss $L_{Adversarial}^{D}$ is defined as the combination of generator loss, where the output from the generator is regarded as fake, and the MSE error of the real GT frame $f_{t}^{B}$ is regarded as real:

(3) $$L_{Adversarial}^{G}=L_{GAN}^{G}=MSE(D(G\left(f_{t}^{A}\right)),\;1)$$

(4) $$L_{Adversarial}^{D}=\frac{1}{2}MSE\left(D\left(G\left(f_{t}^{A}\right)\right),\;0\right)+\frac{1}{2}MSE\left(D\left(f_{t}^{B}\right),\;1\right)\;$$

#### 2.3.2. Perceptual Loss

Perceptual loss measures the difference between the feature representations of the generated image and the ground truth image. It encourages the generated image to match the target image, not only in terms of pixel-wise differences but also in terms of high-level visual features previously learned from classifying comprehensive and diverse datasets. We also used perceptual loss [26], which uses features from different depths of a pre-trained VGG19 [35] network. The use of perceptual loss enables the network to learn turbulence-related features that change the visual style and context of the predicted frame ${\hat{f}}_{t}^{B}$ with respect to the clean frame $f_{t}^{B}$ while maintaining the innovative capabilities of the GAN architecture. Perceptual loss is defined as follows [26]:

(5) $${L_{VGG19}^{G}=L}_{Perceptual}^{G}=L_{Content}^{G}+100\times L_{Style}^{G}$$

where Style loss is defined as:

(6) $$L_{Style}^{G}=\sum_{l\in L}^{}w_{l}\times MSE({gram(\phi_{l}(f}_{t}^{B})),gram(\phi_{l}({\hat{f}}_{t}^{B})))$$

and $w_{l}$ represents the predefined weights for each layer l, as defined in [26], and gram stands for the normalized Gram matrix:

(7) $$gram\left(X\right)=\frac{X^{T}X}{B*C*H*W}$$

where B,C,H,W are the dimension sizes of matrix X. Finally, Content loss is defined as:

(8) $$L_{Content}^{G}=\|{\left.{\phi_{3}(f}_{t}^{B}),\phi_{3}\left({\hat{f}}_{t}^{B}\right)\right\|}_{1}$$

#### 2.3.3. Optical Flow Loss

We aimed to restore videos degraded by atmospheric turbulence (not just restore images, as often carried out), a task that has additional challenges with respect to single-image restoration. While in the former, one needs to ensure temporal consistency between the frames of the output video for it to be well restored; in the latter, only one frame is outputted and validated for spatial deformities. To attend to this particular challenge, we used two measures: first, we used the dense optical flow algorithm from [34] for pre- and post-processing of the given adjacent turbulent frames $f_{t-1}^{A}$, $f_{t}^{A}$ and the predicted restored frames ${\hat{f}}_{t-1}^{B}$, ${\hat{f}}_{t}^{B}$, respectively. The optical flow may not be accurate for the sole purpose of AT restoration but it still holds valuable information about movements and changes in camera settings, such as in the case of zoom and radial movement of the camera, and information about dynamic objects in the scene, though it may be affected by the turbulence-induced movements. Therefore, knowledge of the flow fields in the scene may contribute to the model’s understanding of the temporal behavior of both the scene and turbulence. Secondly, we trained our module to optimize for the Optical Flow loss between the predicted optical flow ${\hat{OF}}_{t}^{B}$ (between ${\hat{f}}_{t-1}^{B}$ and ${\hat{f}}_{t}^{B}$) and the ground truth (GT) flow $OF_{t}^{B}$ (calculated using [34], between real GT frames without turbulence, $f_{t-1}^{B}$ and $f_{t}^{B}$) respectively, using the $L_{1}$ loss, so the model will be penalized for incoherency of movement between timestamps and will, therefore, be encouraged to produce temporally consistent frames with respect to scene dynamics.

(9) $$L_{OF}^{G}={\left\|{OF_{t}^{B},\;\hat{OF}}_{t}^{B}\right\|}_{1}$$

#### 2.3.4. Total Variation Loss

Total Variation (TV) loss [31] is a regularization technique commonly used in image processing and computer vision tasks. It encourages smoothness and reduces noise in the output image by penalizing rapid changes or high-frequency components. The primary motivation behind using TV loss is to preserve the structural integrity of the image while removing noise and unwanted artifacts.

The loss function is defined by taking the sum of the absolute differences between adjacent pixels in the image in both the horizontal and vertical directions, and the final TV loss is the sum of these two components.

(10) $$L_{TV}^{G}=\frac{1}{2N}\sum {\left\|f\left(i,j\right)-f\left(i+1,j\right)\right\|}_{1}+\frac{1}{2N}\sum {\left\|f\left(i,j\right)-f\left(i,j+1\right)\right\|}_{1}$$

where $N=\left(H-1\right)*(W-1)$ and H,W are the height and width of the image, respectively.

#### 2.3.5. Atmospheric Turbulence Loss

This loss was designed specifically for our network. It is an unsupervised loss function fully contained from the networks’ outputs, which uses posterior knowledge of the turbulence from the network’s M&F Unit stage to teach an earlier stage of the network to predict AT flow.

(11) $$L_{AT}^{G}={\left\|{\hat{OF}}_{t}^{AT_{expected}},{\hat{OF}}_{t}^{AT}\;\right\|}_{1}$$

**Figure 7:**
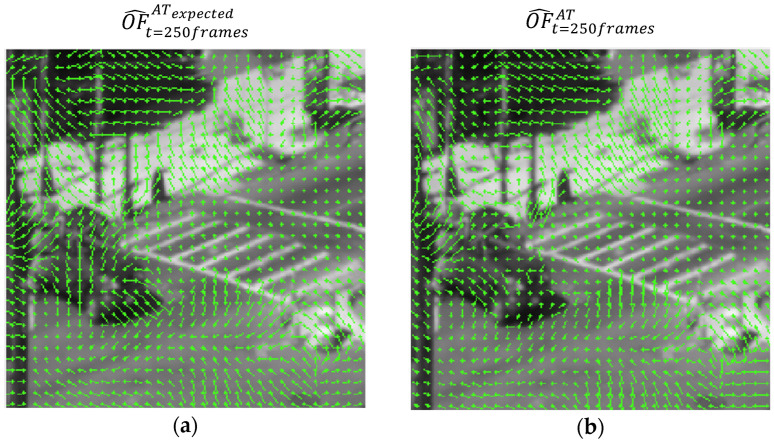
A visual comparison of AT optical flow learning for the AT prediction network (vector field is increased by a factor of 8 for visual purposes), where the green lines represents the optical flow from ${\hat{f}}_{t=249frames}^{B}$ to ${\hat{f}}_{t=250frames}^{B}$ for each 8th pixel in the image. (**a**) The expected AT flow sampled at frame 250, predicted in the output from the AT Prediction Network. (**b**) The calculated AT flow at frame 250, from stage 3 in Figure 1.

The pseudo prediction of the AT flow is used twice: first for the training of the AT Predication Network, which is optimized to predict the current AT flow using an $L_{1}$ loss between the expected and predicted AT flow ${\hat{OF}}_{t}^{AT_{expected}}$, which is the output of the AT Prediction network (Figure 3) and the calculated current flow ${\hat{OF}}_{t}^{AT}$, as can be seen visually in Figure 7. That said, this loss is highly dependent on the optical flow algorithm used since it acts as an optical flow predictor for the AT and is directly affected by its errors.

The second use, as explained before, is in the updating of both auxiliary cells, where the justification for such memory cell comes directly from the quasi-periodic attribute of the turbulence under the thesis that the network can estimate the AT flow over time at different spatial areas of the scene and estimate the correct changes needed to overcome it.

#### 2.3.6. Overall Loss

Finally the complete Loss function is the weighted sum of the individual loss functions, where $\lambda_{GAN}$, $\lambda_{VGG19}$, $\lambda_{OF}$, $\lambda_{AT}$ and $\lambda_{TV}$ are the corresponding weights for $L_{GAN}^{G}$, $L_{VGG19}^{G}$, $L_{OF}^{G}$, $L_{AT}^{G}$ and $L_{TV}^{G}$, respectively. Their values are presented in Section 3.2.

(12) $$L_{ATVR}^{G}=\;\lambda_{GAN}\times L_{GAN}^{G}+\lambda_{VGG19}\times L_{VGG19}^{G}\;+\lambda_{OF}\times L_{OF}^{G}+\lambda_{AT}\times L_{AT}^{G}+\lambda_{TV}\times L_{TV}^{G}$$

## 3. Results

Our algorithm was first trained and evaluated on our synthetic dataset, followed by testing with real AT-degraded data. To assess and compare the performance of our model with other state-of-the-art algorithms, and since no video-to-video method with published code could be found, we used AT image-to-image restoration algorithms like AT-Net [9] and BATUD [5] and the AT sequence-to-image restoration algorithm CLEAR [12] for our AT restoration comparison. Also, we compared our work with the image restoration model MPRNet [36] to see if such a general image restoration model can outperform dedicated AT restoration models.

In order to compare the different methods, each method was trained and evaluated with our dataset while using the published hyperparameters and code. For CLEAR [12], which is a sequence-to-image model, we followed the authors’ work in [37] and used a sequence of five reference frames for each time stamp.

The performance of the different methods on the synthetic data is evaluated in terms of Peak Signal-to-Noise Ratio (PSNR) and Structural Similarity Index (SSIM), which are very common metrics used for evaluating image/video denoising and restoration tasks, as can be seen in [5,12,19] and more.

### 3.1. Dataset and Data Preparation

In the absence of a formal atmospheric turbulence benchmark, over the years, different methods for atmospheric turbulence simulation and synthesis have been proposed. Some do not rely on a physical model, like the ones used in [8,9,19], which rather use random blurring kernels and random motion fields while following Equation (1). Others use physical modeling of the turbulence, such as [32], which takes into account the distance between the camera and the object and turbulence strength. After studying the different methods for AT simulation, we used the method suggested in [32] to create our dataset.

Our training and validation dataset was created by gathering different kinds of videos from online sources, which were used for the creation of our synthetic dataset. These videos had little to no visible turbulence interference across different scenes, such as animals in the wild versus people walking in a crowded street and different object dynamics, such as horizontal and vertical movements. Moreover, we added videos containing changes in camera settings like zoom in and zoom out to address various changes that can be encountered when filming long-range videos. 

For a test dataset, we used the publicly available CDnet 2014 dataset [38], which contains 11 video categories with four to six video sequences in each category. However, we only included videos captured outdoors under clear weather conditions for our synthetic dataset. This ensured that the videos were free from additional disturbances and provided a suitable foundation for generating synthetic atmospheric turbulence.

Furthermore, the CDnet dataset [38] includes four real turbulent videos that exhibited visible atmospheric turbulence effects. These videos were used for the visual assessment and benchmarking of our algorithm’s performance on real AT videos. Dataset division, along with the number of videos and frames used in training, validation, and testing, respectively, is detailed in Table 2.

To create synthetic AT videos, we resized the video frames to 256 × 256 pixels and converted them to grayscale (0–255), and then we calibrated the distance, aperture diameter and turbulence degree for all the videos and inserted them frame by frame using the method proposed by [38]. All of the synthetic data were created with a mean wavelength of 0.525 µm, and the other parameters that correspond to different imaging conditions are detailed in Table 3.

### 3.2. Training Details

The end-to-end design was implemented in Pytorch, and the training was performed using a single GeForce RTX 2060 Super GPU. In training, a batch of four randomly picked synthesized AT sequences of 10 frames each and their corresponding GTs are drawn from the training set. The frames are normalized to the range of [−1, 1]. During training, we used the Adam solver [39] with the hyperparameters of β1 = 0.9 and β2 = 0.999 to perform one step of the update on the discriminator and then one step on the generator for each predicted frame in the sequence. After going through all the frames in the sequence and before inserting new frames from different videos, initialization of the recurrent cells in the generator is performed to prevent the generator from learning unreal scenarios. The learning rate is initially set at 0.0005, and an “On-Plateau” learning rate scheduler is applied with a patience parameter of 50 validation iterations, a division factor of 0.5 and a threshold of 0.01. For the hyperparameters in the loss function (Equation (12)), we empirically set $\lambda_{VGG19}$ = 10 and $\lambda_{OF}=\lambda_{GAN}=\lambda_{AT}={\;\lambda }_{TV\;}=1$. The empirical setting of the lambda parameters stemmed from various experiments conducted during the research, where different settings for each loss were examined. We found that having the $\lambda_{VGG19}$ set to a relatively high value w.r.t enabled the rest of the loss functions, encouraged the generator to learn better and quicker, and yielded a more stable GAN training while aiding with other loss convergences.

### 3.3. Testing Details

The testing procedure contained both synthetic data, for which we have GT and can provide quantitative results, and real-world turbulence distorted videos, for which no GT could be provided and, thus, a qualitative comparison of the different methods can be inspected visually.

### 3.4. Results on Synthetic Data

**Figure 8:**
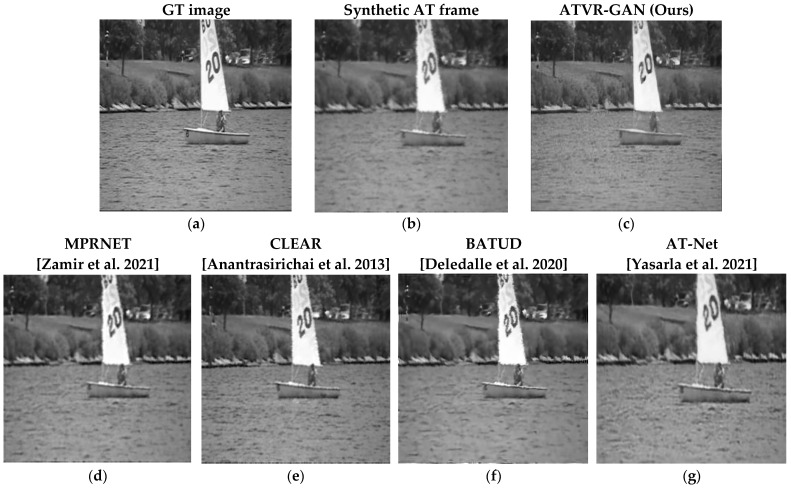
Results of comparison of the different methods on the “Boats” from CDnet 2014 dataset [38] with synthetic AT parameters from set 6 Table 3. (**a**) GT frame, (**b**) GT frame induced synthetically with AT yielding the synthetic AT frame. (**c**) Our results (**d**–**g**) are [5,9,12,36] reconstructed frames, respectively. Video avilable at: https://www.youtube.com/watch?v=Lt0R5R6rKoU, accessed on 16 September 2023.

The comparative results corresponding to different methods used in relation to synthetic data are summarized in Table 4, where higher PSNR and SSIM correspond to a better quality in terms of the reconstructed videos. Visual examples for the second row in Table 4 are shown in Figure 8 from the “boats” video. As can be seen from Figure 8 and Table 4, ATVR-GAN outperforms these state-of-the-art image-restoration and AT mitigation methods. In particular, ATVR-GAN, which utilizes prior knowledge of the turbulence from previous frames, manages to produce better images by 10.24% and 17.39% over the input frames and by 4.16% and 5.73% over the second-best algorithm [9], with regard to PSNR and SSIM, respectively. Moreover, we can see that our model was able to overcome harsh displacements, as can be seen when examining straight lines in the image, like the boat’s sail. Additionally, our algorithm was able to reconstruct fine features of the image, like the bushes in the background, which are absent from the input AT frame.

As can be seen, ATVR-GAN, which utilizes knowledge of the nature of AT motion and data from previous time stamps, both architecturally and via a dedicated cost function, is able to generate sharper and clearer frames while counting for previous frames and, thus, generates more coherent video frames. As can be seen in Figure 8, our model can improve the simulated AT conditions.

### 3.5. Results on Real Data

**Figure 9:**
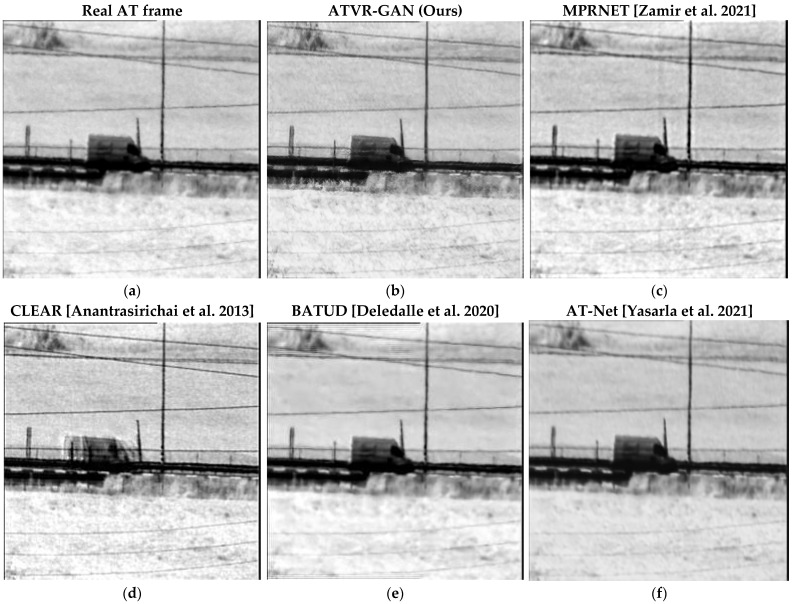
Comparison of reconstructed frames using the above mentioned models on a real AT frame. (**a**) Real AT frame; (**b**) our results; (**c**–**f**) are [5,9,12,36] reconstructed frames, respectively. Video avilable at: https://www.youtube.com/watch?v=Kew7y8vndjo, accessed on 16 September 2023.

The performance of the described methods was also evaluated against real-world turbulence-distorted videos. Figure 9 presents the reconstruction results of the compared methods on a real-world distorted video from the CDnet 2014 dataset [38], where the AT degradation is assessed to be of low distortion and medium blur. In addition to turbulence, it may also contain particles in the atmosphere that sometimes cause blur. Additionally, this video contains a dynamic scene of moving cars without a change in camera position. Using a qualitative visual comparison of the different methods, it can be observed that ATVR-GAN was able to restore the real-world video frame while preserving the original details.

## 4. Conclusions

We proposed a method termed ATVR-GAN to address the problems that arise during the reconstruction of a video damaged by atmospheric turbulence in long-distance imaging. This is a problem entailing both geometric deformations and blur in both time as well as in spatial domains. We took on the challenge of video-to-video AT restoration, which has been the least studied problem over the last decade compared to image-to-image and sequence-to-image restoration models. Our model was specially designed to tackle the AT problem using a specialized generator architecture that utilizes the time domain as well as custom loss functions that drive the network to predict the current flow of the turbulence and counts for its quasiperiodic nature. We showed that our model can generalize to unseen or closely resembled scenes, which shows the model’s capabilities to learn the nature of AT. Our model outperformed the state-of-the-art methods in terms of generating improved frames and video sequences with less blur and deformation on real and synthetic data. Nevertheless, further work should be carried out to better generalize the model for the variety of severely turbulence-degraded videos.

## Figures and Tables

**Table 1:** The architecture of the hidden blocks in Memory and Flow Extraction (M&F) Unit.

Input Dimension *	Layer	Output Dimension
{256, 256, 1, 4}	Conv2D ($Kernal=\left(3\times 3\right),\;stride=1)$ Instance Normalization ReLU	{256, 256, 1, 4}
{256, 256, 1, 4}	Conv2D ($Kernal=\left(3\times 3\right),\;stride=1)$ Instance Normalization	{256, 256, 1, 2}

* The dimensions in the table are set as {height, width, channels, and dimensions}.

**Table 2:** Properties of the real and synthetic datasets.

Videos/Number of Frames	Training	Validation	Testing
Synthetic AT dataset	10 different videos per set (80 videos) ~100,000 frames	3 different videos per set (24 videos) ~30,000 frames	7 different videos with setting from set6 and set1 (14 videos) ~11,000 frames
Real AT dataset			4 videos

**Table 3:** Synthetic dataset simulation hyperparameters.

Simulations Sets	Propagation $\mathrm{Length}\;(L)\;\left[m\right]$	Refractive $\mathrm{Index}\;\mathrm{Structure}\;(C_{n}^{2}$$)\;\left[m^{-\frac{2}{3}}\right]$	Fried $\mathrm{Parameter}\;(r0)\;\left[m\right]$	Aperture $\mathrm{Diameter}\;(D)\;\left[m\right]$
set1	4000	$1.1\times 10^{-17}$	1	0.1
Set2	4000	$0.35\times 10^{-17}$	2	0.1
Set3	4000	$0.18\times 10^{-17}$	3	0.1
Set4	4000	$0.11\times 10^{-17}$	4	0.1
Set5	1000	$0.65\times 10^{-14}$	0.05	0.2
Set6	1500	$0.43\times 10^{-14}$	0.05	0.2
Set7	2000	$0.32\times 10^{-14}$	0.05	0.2
Set8	2500	$0.26\times 10^{-14}$	0.05	0.2

**Table 4:** Quantitative comparison results in terms of PSNR/SSIM on synthetic datasets, where the best results are marked in bold, and the second best are underlined.

Dataset/ Degradation Level	AT Raw Input	CLEAR [12]	MPRNET [36]	BATUD [5]	AT-Net [9]	Ours ATVR-GAN
D = 0.1|L = 4000|r0 = 1 $C_{n}^{2}=1.1\times 10^{-17}$	20.98/ 0.586	20.64/ 0.571	21.55/ 0.635	20.02/ 0.567	22.77/ 0.692	**23.96**/ **0.741**
D = 0.2|L = 1500|r0 = 0.05 $C_{n}^{2}=0.43\times 10^{-14}$	22.58/ 0.703	22.00/ 0.692	23.21/ 0.753	21.96/ 0.685	23.34/ 0.738	**24.05**/ **0.770**
Average Test Scores	21.78/ 0.644	21.32/ 0.631	22.38/ 0.694	20.99/ 0.626	23.05/ 0.715	**24.01**/ **0.756**

## Data Availability

Data are available in a publicly accessible repository. The data presented in this study are openly available under the reference number [38].
